# Prognostic Impact of Cardiovascular Injuries for Patients with Respiratory Isolated Chest Trauma

**DOI:** 10.1093/icvts/ivaf266

**Published:** 2025-11-06

**Authors:** Shuji Mishima, Kimihiro Shimizu, Hitoshi Igai, Ichiro Okada, Toru Takiguchi, Makoto Aoki, Youichi Yanagawa, Daizoh Saito, Kenji Suzuki, Morihito Okada, Masayuki Chida, Ichiro Yoshino

**Affiliations:** Division of General Thoracic Surgery, Department of Surgery, Shinshu University School of Medicine, Matumoto, Nagano, 390-8621, Japan; Division of General Thoracic Surgery, Department of Surgery, Shinshu University School of Medicine, Matumoto, Nagano, 390-8621, Japan; Department of General Thoracic Surgery, Japanese Red Cross Maebashi Hospital, Maebashi, Gunma, 371-0811, Japan; Department of Emergency and Critical Care Medicine, Nippon Medical School Chiyoda-ku, Tokyo, 101-8309, Japan; Department of Emergency and Critical Care Medicine, Nippon Medical School Chiyoda-ku, Tokyo, 101-8309, Japan; Department of Emergency Medicine and Critical Care Medicine, Advanced Medical Emergency and Critical Care Center, Japanese Red Cross Maebashi Hospital, Maebashi, Gunma, 371-0811, Japan; Department of Acute Critical Care Medicine, Shizuoka Hospital, Juntendo University Izunokuni, Shizuoka, 410-2211, Japan; Department of Traumatology and Critical Care Medicine, National Defense Medical College Tokorozawa, Saitama, 359-8513, Japan; Graduate School of Emergency Medical System, Kokushikan University, Tama, Tokyo, 206-0025, Japan; Department of General Thoracic Surgery, Juntendo University School of Medicine, Bunkyo-ku, Tokyo, 113-0033, Japan; Department of Surgical Oncology, Research Institute for Radiation Biology and Medicine, Hiroshima University, Hiroshima, Hiroshima, 734-0037, Japan; Department of General Thoracic Surgery, Dokkyo Medical University, Mibu, Tochigi, 321-0293, Japan; International University of Health and Welfare, Narita, Chiba, 286-0048, Japan

**Keywords:** cardiovascular injury, isolated chest trauma, Japan Trauma Data Bank, respiratory injury

## Abstract

**Objectives:**

This study assessed the prognostic impact of cardiovascular injuries in patients with isolated chest trauma primarily involving the respiratory system.

**Methods:**

We retrospectively reviewed the Japan Trauma Data Bank records (2004-2019). Patients with isolated chest trauma were categorized into the respiratory or cardiovascular injury group according to the highest abbreviated injury scale score. The effect of cardiovascular injuries in the respiratory injury group was analysed using a multivariable logistic regression analysis.

**Results:**

Among the 8048 patients in the respiratory injury group, those with minor cardiac injury had a higher mortality rate than those without (15% vs 7%; *P *= .027); those with severe vascular injury (most commonly thoracic aorta) had a 76% mortality rate. The multivariable analysis indicated older age (adjusted odds ratio [adjOR]: 1.01, 95% CI: 1.00-1.01, *P *= .016), penetrating injury (adjOR: 2.19, 95% confidence interval [CI]: 1.40-3.43, *P *= .002), higher new injury severity score (adjOR: 3.89, 95% CI: 3.16-4.78, *P *< .001), coexistence of cardiac (adjOR: 2.68, 95% CI: 1.51-4.76, *P *< .001) or vascular injuries (adjOR: 3.36, 95% CI: 1.93-5.83, *P *< .001), and tracheobronchial injuries (adjOR: 2.10, 95% CI: 1.15-3.82, *P *= .015) with the highest abbreviated injury scale scores were significantly associated with increased odds of in-hospital mortality.

**Conclusions:**

Minor cardiac or severe vascular injuries significantly increased mortality in patients with isolated chest trauma primarily involving the respiratory system. Assessment of both respiratory and coexisting cardiovascular injuries is essential for clinical management.

## Graphical Abstract

**Figure:**
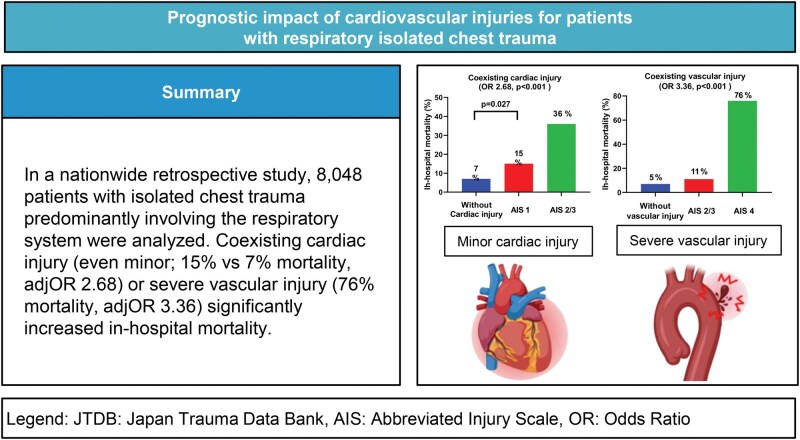

## INTRODUCTION

Chest trauma can cause injury to vital organs, especially those in the cardiovascular and respiratory systems, and is a major cause of trauma-related death. In Japan, moderate or severe chest trauma accounts for approximately 10% of the cases, with mortality rates reported between 2.8% and 50%.^1^ It is the second most common cause of trauma-related death after head and neck trauma.^1^ Blunt trauma is far more common than penetrating trauma and is directly responsible for up to 25% of trauma deaths.^2^

Cardiac and thoracic vessel injuries have high mortality rates. Blunt cardiac injury mortality rates range from 14.5% to 32%,^3^^,^^4^ and can reach 76% for penetrating injuries.^5^ Thoracic aortic injuries are the leading cause of death after head trauma in blunt chest trauma; 32%-50% of patients die within 24 hours.^6–8^

In contrast, the mortality rates for respiratory injuries (lung, trachea, bronchus, chest wall) depend on the presence of associated injuries; isolated pulmonary contusion has a lower mortality than when accompanied by other thoracic injuries.^9^

Although the prognostic impact of cardiovascular injury is recognized, few studies have addressed its effect when coexisting with respiratory trauma, particularly when respiratory injury is predominant. In addition, whether minor cardiovascular injuries affect survival outcomes for these patients has not been determined.

This study aimed to clarify the characteristics and prognostic effect of coexisting cardiovascular injuries of varying severities in patients with isolated chest trauma predominantly involving the respiratory system, using a nationwide Japanese trauma database.

## METHODS

### Data resources

This retrospective study used data from the nationwide Japan Trauma Data Bank (JTDB), which includes demographic, injury, transport, vital signs, abbreviated injury scale (AIS) scores, interventions, and clinical outcome information (**Table S1**). The AIS scores are coded according to AIS 90, which were updated in 1998,^10^ and entered online by trained physicians. The JTDB is managed by the Japan Association for the Surgery of Trauma and monitored for data quality by the Japanese Association for Acute Medicine.

### Data collection and study design

This retrospective study using the JTDB was approved by the Institutional Review Board of Shinshu University Hospital (ID 6185, June 24, 2024), with informed consent waived by opt-out. The study was conducted in accordance with the WMA Declaration of Taipei and the WMA Declaration of Helsinki.

We reviewed 361 706 JTDB records (2004-2019) and identified 12 866 patients with isolated chest trauma (top 3 AIS scores for chest). We collected patient demographics and clinical information from the JTDB including age, sex, injury year, means of transport, injury mechanism, injured regions with AIS scores, new injury severity score (NISS), admission vital signs (systolic blood pressure, heart rate, respiratory rate, Glasgow coma scale), blood transfusion within 24 hours of admission, and discharge status. Additional data on emergency interventions (such as intubation) and urgent operations were obtained from supplementary sources.

A total of 3387 patients with missing or unknown data for those variables (except emergency intervention and urgent operation) and 4 patients with burn injuries were excluded. We also excluded patients classified as having only “skin/subcutaneous” injuries (without organ injury), “penetrating” injuries only (without specifying the injured organ), “extensive contusion of the entire chest” (all assigned an AIS score of 6 [fatal] without specification), and those whose highest AIS score corresponded to oesophageal or thoracic duct injuries (not categorized as cardiovascular or respiratory injuries).

After applying these criteria, 8865 patients remained and were categorized into 2 groups according to the region with the highest AIS score: (1) the respiratory injury group, comprising those whose highest AIS scores were for respiratory system injuries (trachea/bronchus, lung, pleura, thoracic cavity [pneumothorax/haemothorax], diaphragm, ribs, sternum); and (2) the cardiovascular injury group, comprising those whose highest AIS scores were for cardiovascular system injuries.

**Figure 1.**
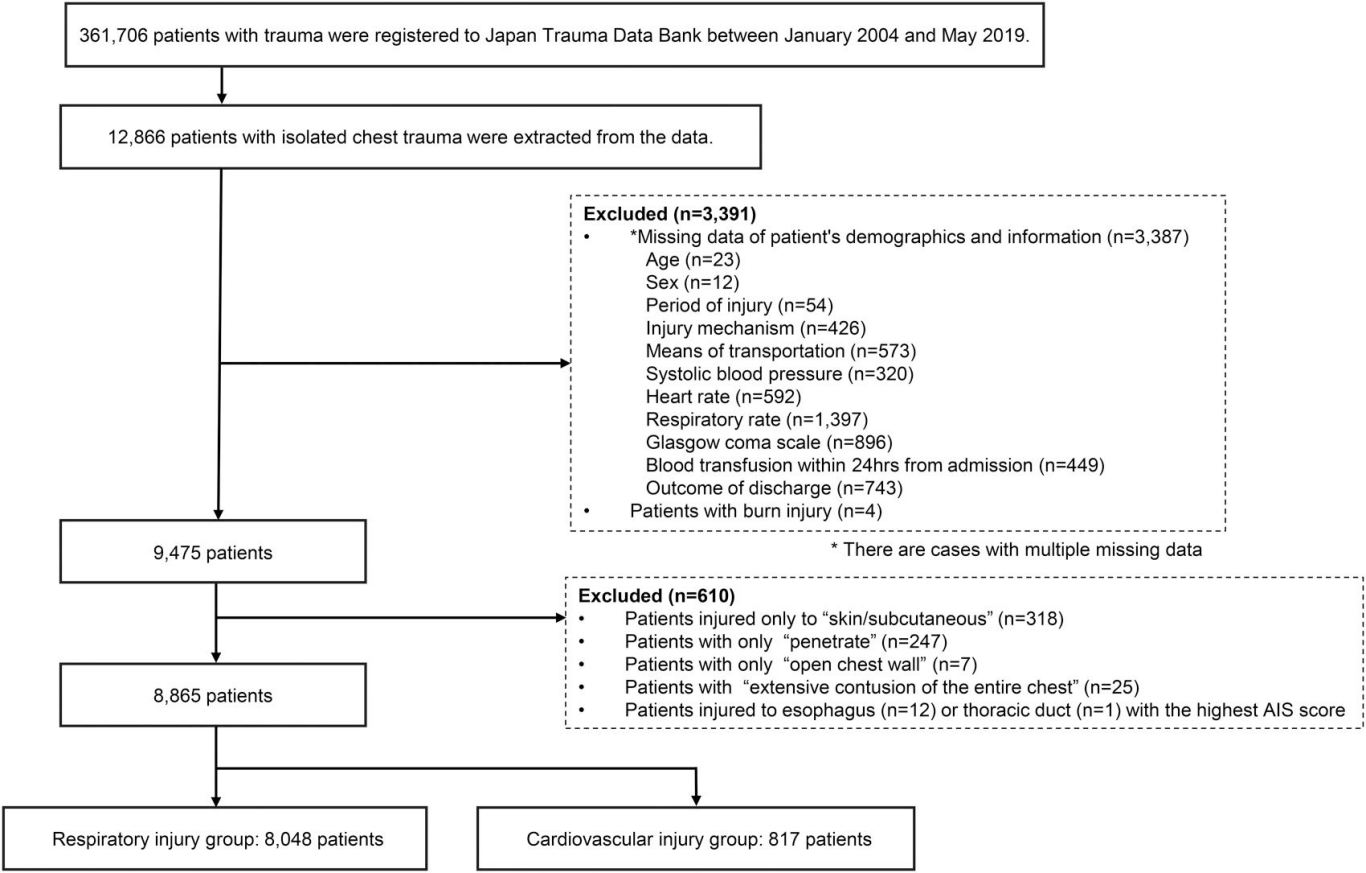
Study Cohort Diagram

If the highest AIS score was assigned to “open chest injury,” “penetrating injury,” or “skin/subcutaneous injury,” the second highest AIS score was used for classification because these categories do not specify a particular organ. If the highest AIS scores for respiratory and cardiovascular injuries were identical, the patient was placed in the cardiovascular injury group. **Figure 1** displays the study diagram.

### Variable definitions and modifications

Several variables were redefined or modified in this study. “Signs of life on admission” was defined as absent if any of the following admission vital signs were obtained: systolic blood pressure (SBP) 0 mmHg, heart rate (HR) 0 bpm, respiratory rate (RR) 0 breaths/min, or Glasgow coma scale score 3. Otherwise, “signs of life” were considered present. For the trend analysis, the injury year was categorized into 3 periods: 2004-2008, 2009-2013, and 2014-2019.

### Statistical analysis

In-hospital mortality was the primary outcome. Patient survival status was determined from the discharge codes; “dead” was coded directly and “alive” was determined from codes indicating discharge or transfer to another hospital.

Continuous variables are presented as medians (interquartile ranges) and categorical variables as percentages. Normality was assessed using the Shapiro-Wilk test. For group comparisons, the Student’s t or Mann-Whitney U test was used for continuous variables, and the chi-square or Fisher’s exact test was used for categorical variables. The respiratory injury group mortality rates were compared across the 3 periods using the Cochran-Armitage trend test.

The univariable and multivariable logistic regression analyses identified factors associated with in-hospital mortality. Age and sex were included in all models; other variables with a univariable *P *< .25 were also selected. Rib and sternal injuries were combined in the category “chest wall injuries.” Model fit was assessed using the Hosmer-Lemeshow test and multicollinearity by variance inflation factors. The results are reported as odds ratios (ORs) and 95% confidence intervals (CIs). Missing data were not imputed because the variables with missing values (except age and sex) were excluded from the multivariable analysis. Patients missing age or sex information were rare and were excluded.

All tests were 2-tailed, and a *P*-value of < .05 was considered significant. All statistical analyses were performed using IBM SPSS Statistics version 27 (IBM Corp., Armonk, NY, USA).

## RESULTS

### Respiratory and cardiovascular injury group characteristics

Of the 8865 patients, the 8048 with isolated chest trauma comprised the respiratory injury group and 817 comprised the cardiovascular injury group (**Figure 1**). The patients in the respiratory group were older (median 63 vs 57 years, *P *< .001) and more likely to have sustained blunt trauma (96% vs 74%, *P *< .001). The cardiovascular group had higher median NISS (36 vs 13, *P *< .001), worse admission vital signs, a lower rate for signs of life (38% vs 94%, *P *< .001), and a higher in-hospital mortality rate (70% vs 7%, *P *< .001) (**Table 1**). Notably, in each group, at least 90% of deaths occurred within 30 days.

**Table 1.** Comparison of Patient Characteristics between the Respiratory Injury Group and the Cardiovascular Injury Group

Characteristics	Respiratory injury group	Cardiovascular injury group	
	*n *= 8048	*n *= 817	*P*-value
Age	63 (45-76)	57 (39-70)	<.001
Sex			.029
Male	5868 (73)	566 (69)	
Female	2180 (27)	251 (31)	
Period of injury			<.001
2004-2008	785 (10)	125 (15)	
2009-2013	2758 (34)	328 (40)	
2014-2019	4505 (56)	364 (45)	
Injury mechanism			<.001
Penetrate	318 (4)	209 (26)	
Blunt	7730 (96)	608 (74)	
Means of transport			<.001
Ambulance	6637 (83)	749 (92)	
Helicopter	512 (6)	63 (7)	
Walk-in/private vehicle	794 (10)	4 (0.5)	
Other	105 (1)	1 (0.1)	
The highest AIS score			<.001
1—minor	175 (2)	14 (2)	
2—moderate	547 (7)	15 (2)	
3—serious	4264 (53)	86 (11)	
4—severe	2581 (32)	115 (14)	
5—critical	481 (6)	299 (36)	
6—fatal	0 (0)	288 (35)	
NISS	13 (9–18)	36 (25–43)	<.001
Vital signs on admission			
SBP (mmHg)	138 (118-158)	0 (0-91)	<.001
HR (per minute)	82 (70-95)	0 (0-90)	<.001
RR (bpm)	20 (18–25)	0 (0-20)	<.001
GCS	15 (15–15)	3 (3–12)	<.001
Signs of life on admission			<.001
Presence	7594 (94)	309 (38)	
Absence	454 (6)	508 (62)	
Outcome at discharge			<.001
Alive	7457 (93)	242 (30)	
Discharged home	5780 (78)	148 (61)	
Transfer to another facility	1599 (21)	90 (37)	
Others	63 (1)	3 (1)	
Unknown	15 (0.2)	1 (0.4)	
Dead	591 (7)	575 (70)	
Within 30 days of admission	534 (90)	540 (94)	

Data are expressed as the number of patients (%) or median (IQR).

Abbreviations: AIS: abbreviated injury scale; GCS: Glasgow coma scale; HR: heart rate; NISS: new injury severity score; RR: respiratory rate; SBP: systolic blood pressure.

Urgent interventions were more common in the cardiovascular group, including blood transfusion within 24 hours (34% vs 6%), cardiac massage (82% vs 6%), tracheal intubation (66% vs 9%), vasopressor use (34% vs 3%), and urgent surgery (59% vs 9%) (all *P *< .001) (**Table S2**).

The in-hospital mortality rates in the respiratory group significantly decreased over time (12% in 2004-2008, 8.8% in 2009-2013, 5.6% in 2014-2019, *P *< .001) (**Figure S1A**), whereas no significant trend was observed for those in the cardiovascular group (*P *= .6) (**Figure S1B**).

### Comparison between survivors and non-survivors in the respiratory injury group

**Table 2** presents a comparison of clinical characteristics between survivors (*n* = 7457) and non-survivors (*n* = 591) in the respiratory injury group. Non-survivors more frequently had critical injuries (AIS 5: 44% vs 3%, *P *< .001), higher median NISS (25 vs 10, *P *< .001), and worse vital signs at admission (all *P *< .001). Injuries to the trachea/bronchus (3% vs 1%, *P *< .001), lungs (26% vs 22%, *P *= .015), and thoracic cavity (15% vs 5%, *P *< .001) were more common among non-survivors. Notably, among the non-survivors with cardiac injury, 44% (8 patients) died despite the injury being classified as AIS 1 (minor).

**Table 2.** Comparison between Survivors and Non-survivors in the Respiratory Injury Group

Characteristics	Survivors	Non-survivors	*P*-value
	*n *= 7457	*n *= 591	
Age in years	62 (46-75)	64 (44-77)	.3
Sex			.7
Males	5441 (72)	427 (73)	
Females	2016 (28)	164 (27)	
Period of injury			<.001
2004-2008	690 (9)	95 (16)	
2009-2013	2516 (34)	242 (41)	
2014-2019	4251 (57)	254 (43)	
Injury mechanism			.18
Penetrate	288 (4)	30 (5)	
Blunt	7169 (96)	561 (95)	
Means of transport			<.001
Ambulance	6105 (81)	532 (90)	
Helicopter	549 (7)	53 (1)	
Walk-in/private vehicle	789 (11)	5 (0.8)	
Other	104 (1)	1 (0.2)	
Highest AIS score			<.001
1—minor	170 (2)	5 (1)	
2—moderate	534 (7)	13 (2)	
3—serious	4130 (55)	134 (23)	
4—severe	2402 (32)	179 (30)	
5—critical	221 (3)	560 (44)	
6—fatal	0 (0)	0 (0)	
NISS	10 (9–16)	25 (16–25)	<.001
Vital signs on admission			
SBP (mmHg)	140 (121-160)	0 (0-40)	<.001
HR (per minute)	83 (72-96)	0 (0-70)	<.001
RR (bpm)	21 (18–25)	0 (0-13)	<.001
GCS	15 (15–15)	3 (3–3)	<.001
Signs of life on admission			<.001
Presence	7438 (99)	156 (26)	
Absence	19 (0.3)	435 (74)	
Most severely injured region			
Trachea/bronchus	58 (1)	21 (3)	<.001
Lungs	1769 (22)	167 (26)	.015
Pleura	107 (1)	10 (2)	.7
Thoracic cavity	582 (7)	95 (15)	<.001
Diaphragm	49 (1)	6 (1)	.4
Ribs	5390 (66)	335 (52)	<.001
Sternum	257 (3)	5 (1)	<.001
Coexistence of cardiovascular injury			
Cardiac injuries	63 (1)	18 (3)	<.001
Vascular injuries	46 (1)	21 (4)	<.001
Cardiac injury details			
AIS score			.006
1—minor	45 (72)	8 (4)	
2—moderate	11 (17)	2 (12)	
3—serious	7 (11)	8 (44)	
4—severe	0 (0)	0 (0)	
5—critical	0 (0)	0 (0)	
6—fatal	0 (0)	0 (0)	
Region			
Myocardium	53 (84)	14 (77)	–
Pericardium	10 (16)	4 (23)	–
Vascular injury details			
AIS score			<.001
1—minor	0 (0)	0 (0)	
2—moderate	30 (65)	0 (0)	
3—serious	11 (24)	5 (24)	
4—severe	5 (11)	16 (76)	
5—critical	0 (0)	0 (0)	
6—fatal	0 (0)	0 (0)	
Region			
Thoracic aorta	4 (9)	8 (38)	-
Brachiocephalic artery	1 (2)	0 (0)	-
Pulmonary artery	0 (0)	4 (19)	-
Pulmonary vein	0 (0)	3 (14)	-
Subclavian artery	1 (2)	4 (19)	-
Subclavian vein	1 (2)	0 (0)	-
Superior vena cava	0 (0)	1 (5)	-
Other arteries	36 (78)	1 (5)	-
Other veins	3 (7)	0 (0)	-

Data are expressed as the number of patients (%) or median (interquartile range).

Abbreviations: AIS: abbreviated injury scale; GCS: Glasgow coma scale; HR: heart rate; NISS: new injury severity score; RR: respiratory rate; SBP: systolic blood pressure.

### Characteristics by severity of co-existing cardiac or vascular injury

**Tables 3 and 4** show the outcomes by severity for coexisting cardiovascular injuries in the respiratory injury group. Patients with minor cardiac injuries (AIS 1) had significantly higher mortality than those without cardiac injury (15% vs 7%, *P *= .027) (**Table 3**). Severe vascular injuries (AIS 4), mainly involving the thoracic aorta, were associated with a remarkably high mortality rate (76%) (**Table 4**).

**Table 3.** Patient Characteristics Based on the AIS Score of Coexisting Cardiac Injuries

Characteristics	Without cardiac injury	AIS 1	AIS 2/3
	*n *= 7967	*n *= 53	*n *= 28
Injury mechanism			
Penetrate	297 (4)	4 (8)	17 (61)
Blunt	7670 (96)	49 (92)	11 (39)
Maximum AIS score of respiratory injuries	3 (3–4)	3 (3–4)	4 (4–5)
Region of the most severe respiratory injury			
Trachea and bronchus	79 (1)	0 (0)	0 (0)
Lungs	1904 (22)	16 (27)	16 (57)
Pleura	117 (1)	0 (0)	0 (0)
Thoracic cavity	664 (8)	9 (15)	4 (14)
Diaphragm	49 (0.6)	1 (2)	5 (18)
Ribs	5693 (65)	25 (42)	6 (21)
Sternum	253 (3)	9 (15)	0 (0)
Region of cardiac injuries			
Myocardium	–	53 (100)	14 (50)
Pericardium	–	0 (0)	14 (50)
Outcome at discharge			
Alive	7394 (93)	45 (85)	18 (64)
Dead	573 (7)	8 (15)^a^	10 (36)^b^
Within 30 days of admission	518 (90)	7 (88)	9 (90)

Data are expressed as the number of patients (%) or median (IQR).

a Significant difference between the patients without cardiac injury and those with AIS 1 cardiac injury (*P* = .027).

b Significant difference between the patients without cardiac injury and those with AIS 2/3 cardiac injury (*P* < .001).

Abbreviation: AIS: abbreviated injury scale.

**Table 4.** Patient Characteristics Based on the AIS Score of Coexisting Vascular Injuries

Characteristics	Without vascular injury	AIS 2/3	AIS 4
	*n *= 7981	*n *= 46	*n *= 21
Injury mechanism			
Penetrate	299 (4)	15 (33)	4 (19)
Blunt	7682 (96)	31 (67)	17 (81)
Maximum AIS score of respiratory injuries	3 (3–4)	4 (3–4)	5 (5–5)
Region of the most severe respiratory injury			
Trachea and bronchus	77 (1)	0 (0)	2 (9)
Lungs	1909 (22)	19 (38)	8 (36)
Pleura	117 (1)	0 (0)	0 (0)
Thoracic cavity	667 (8)	7 (14)	3 (14)
Diaphragm	54 (1)	1 (2)	0 (0)
Ribs	5693 (65)	23 (46)	9 (41)
Sternum	262 (3)	0 (0)	0 (0)
Region of vascular injury			
Thoracic aorta	–	0 (0)	12 (57)
Brachiocephalic artery	–	1 (2)	0 (0)
Pulmonary artery	–	1 (2)	3 (14)
Pulmonary vein	–	1 (2)	2 (10)
Subclavian artery	–	1 (2)	4 (19)
Subclavian vein	–	1 (2)	0 (0)
Superior vena cava	–	1 (2)	0 (0)
Other arteries	–	37 (80)	0 (0)
Other veins	–	3 (7)	0 (0)
Outcome at discharge			
Alive	7411 (93)	41 (89)	5 (24)
Dead	570 (7)	5 (11)	16 (76)^a^
Within 30 days of admission	515 (90)	4 (80)	16 (100)

Data are expressed as the number of patients (%) or median (IQR).

a Significant difference between the patients without vascular injury and those with AIS 4 vascular injury (*P* < .001).

Abbreviation: AIS: abbreviated injury scale.

### Univariable and multivariable analyses of risk factors for in-hospital mortality

The multivariable analysis revealed that older age (adjusted OR [adjOR]: 1.01, 95% CI: 1.00-1.01, *P *= .016), penetrating injury (adjOR: 2.19, 95% CI: 1.40-3.43, *P *= .002), higher NISS (adjOR: 3.89, 95% CI: 3.16-4.78, *P *< .001), coexistence of cardiac (adjOR: 2.68, 95% CI: 1.51-4.76, *P *< .001) or vascular injuries (adjOR: 3.36, 95% CI: 1.93-5.83, *P *< .001), and trachea and bronchus injuries (adjOR: 2.10, 95% CI: 1.15-3.82, *P *= .015) with the highest AIS scores were significantly associated with increased odds of in-hospital mortality. In contrast, injuries to lungs (adjOR: 0.49, 95% CI: 0.37-0.67, *P *< .001), and chest wall (adjOR: 0.28, 95% CI: 0.20-0.38, *P *< .001) with the highest AIS scores were significantly associated with decreased odds of in-hospital mortality (**Table 5**). The model demonstrated adequate calibration (Hosmer-Lemeshow test, *P *= .095) without substantial multicollinearity (all variance inflation factors < 3.2).

**Table 5.** Univariable and Multivariable Analyses for in-Hospital Mortality

Factors	Univariable			Multivariable		
	OR	95% CI	*P*-value	adjOR	95% CI	*P*-value
Age in years	1.00	(0.99-1.01)	.3	1.01	(1.00-1.01)	.016
Females (vs males)	0.97	(0.80-1.16)	.7	0.88	(0.73-1.07)	.2
Penetrate (vs blunt)	0.75	(0.51-1.10)	.15	2.19	(1.40-3.43)	.002
NISS ≥ 13	3.69	(3.02-4.49)	<.001	3.89	(3.16-4.78)	<.001
Coexistence of cardiovascular injuries						
Cardiac injuries	3.69	(2.17-6.27)	<.001	2.68	(1.51-4.76)	<.001
Vascular injuries	5.94	(3.52-10.0)	<.001	3.36	(1.93-5.83)	<.001
Most severely injured region						
Trachea and bronchus	4.7	(2.83-7.80)	<.001	2.10	(1.15-3.82)	.015
Lungs	1.27	(1.05-1.53)	.013	0.49	(0.37-0.67)	<.001
Pleura	1.18	(0.62-2.27)	.6			
Thoracic cavity	2.26	(1.79-2.86)	<.001	0.92	(0.65-1.30)	.6
Diaphragm	1.55	(0.66-3.63)	.3			
Chest wall^a^	0.5	(0.42-0.60)	<.001	0.28	(0.20-0.38)	<.001

a Chest wall includes rib and sternum injury.

Abbreviations: adjOR: adjusted odds ratio; AIS: abbreviated injury scale; CI: confidence interval; NISS: new injury severity score; OR: odds ratio.

## DISCUSSION

To our knowledge, this was the first nationwide study in Japan to examine the prognostic impact of coexisting cardiovascular injuries of varying severity on patients with isolated chest trauma predominantly affecting the respiratory system.

The overall in-hospital mortality in the cardiovascular injury group was higher than that reported in previous studies. This difference is likely attributable to the inclusion of patients without signs of life on hospital arrival in our cohort. Notably, among the patients who presented without signs of life, their mortality rate was extremely high (99%), whereas for those with signs of life, their mortality rates were similar to those in other reports.^3–8^

One of the key findings was that for patients in the respiratory injury group even minor cardiac injuries were associated with significantly higher mortality, with a 2-fold increase compared to those patients without such injuries. These minor injuries, such as myocardial contusions with dysrhythmia or wall motion abnormalities,^10^ are often considered to require minimal intervention. In addition, some cardiac injuries may not have been recognized at admission and therefore were classified as “no injury.” The JTDB does not record the timing of a diagnosis. A prior report suggest that delayed recognition of cardiac trauma can lead to irreversible complications such as myocardial infarction.^11^ Given that clinical findings may be misleading, electrocardiography and cardiac enzyme measurements should be routinely obtained from patients with chest trauma, and further evaluations using echocardiography or coronary angiography should be considered when abnormalities are detected.

Among the patients with vascular injuries, the highest mortality rate was observed for those with an AIS score of 4 (76%); thoracic aorta injuries had the highest occurrence (57%). Although recent advancements in medical technology and interventional radiology have contributed to reduced postoperative complications and improved mortality rates for patients with thoracic aorta injuries,^12–16^ our findings underscore the persistent impact of severe vascular injuries on survival.

The multivariable analysis identified tracheobronchial injury as the most severely injured region significantly associated with higher in-hospital mortality. Although rare, these injuries have a high mortality rate^17^ and are frequently underdiagnosed.^18^ To improve detection, computed tomography and bronchoscopy should be performed^19^ as bronchoscopy remains the gold standard for determining injury location and treatment. In contrast, lung and chest wall (rib/sternum) injuries were associated with lower in-hospital mortality when they were the most severe injuries. These injuries are generally not fatal in isolation; however, when they coexist with other significant injuries, prognosis may worsen.^9^^,^^20^^,^^21^ In our cohort, a NISS ≥ 13 was linked to poor outcomes, indicating that overall injury burden, not just the most severely injured site, played an important role. Therefore, although identifying the most severely injured region within the respiratory system may be helpful, it should be interpreted in the context of the patient’s total injury severity.

This study had several limitations. First, it was a retrospective study based on registry data; therefore, it may be subject to selection bias and unmeasured confounding factors. Second, a considerable number of patients were excluded because of missing or incomplete data, which could affect generalizability. Third, facility-level characteristics could not be evaluated because the JTDB does not record hospital type or trauma centre level. Finally, although we summarized emergency interventions, their diversity and complexity precluded detailed analysis of their effects.

## CONCLUSION

In conclusion, minor cardiac and severe vascular injuries significantly worsened survival outcomes for patients with isolated chest trauma. Careful assessment of cardiovascular involvement and identifying the most severely injured respiratory region may help guide early risk stratification and clinical management.

## Supplementary Material

ivaf266_Supplementary_Data

## Data Availability

The data underlying this article were provided by the Japanese Association for the Surgery of Trauma (Trauma Registry Committee) and the Japanese Association for Acute Medicine (Committee for Clinical Care Evaluation) by permission. Data will be shared on request to the corresponding author with permission of these committees.

## References

[1] Toida C , Muguruma T, Gakumazawa M, Shinohara M, Abe T, Takeuchi I. Ten-year in-hospital mortality trends among Japanese injured patients by age, injury severity, injury mechanism, and injury region: a nationwide observational study. PLoS One. 2022;17:e0272573. 35994453 10.1371/journal.pone.0272573 PMC9394834

[2] Edgecombe L , Sigmon DF, Galuska MA, Angus LD. Thoracic trauma: StatPearls; 2025 [updated May 23, 2023]. Accessed 12 September 2025. http://www.ncbi.nlm.nih.gov/books/NBK534843/30521264

[3] Lancey RA , Monahan TS. Correlation of clinical characteristics and outcomes with injury scoring in blunt cardiac trauma. J Trauma. 2003;54:509-515. 12634531 10.1097/01.TA.0000025312.48962.C5

[4] Hanschen M , Kanz KG, Kirchhoff C, et al; TraumaRegister DGU. Blunt cardiac injury in the severely injured—a retrospective multicentre study. PLoS One. 2015;10:e0131362. 26136126 10.1371/journal.pone.0131362 PMC4489656

[5] Dumani S , Ibrahimi A, Likaj E, et al Cardiac trauma. Management strategies short panoramic view. AJTES. 2023;7:1189-1195.

[6] Neschis DG , Scalea TM, Flinn WR, Griffith BP. Blunt aortic injury. N Engl J Med. 2008;359:1708-1716. 18923173 10.1056/NEJMra0706159

[7] von Oppell UO , Dunne TT, De Groot MK, Zilla P. Traumatic aortic rupture: twenty-year metaanalysis of mortality and risk of paraplegia. Ann Thorac Surg. 1994;58:585-593. 8067877 10.1016/0003-4975(94)92270-5

[8] Jamieson WR , Janusz MT, Gudas VM, Burr LH, Fradet GJ, Henderson C. Traumatic rupture of the thoracic aorta: third decade of experience. Am J Surg. 2002;183:571-575. 12034396 10.1016/s0002-9610(02)00851-6

[9] Clark GC , Schecter WP, Trunkey DD. Variables affecting outcome in blunt chest trauma: flail chest vs. pulmonary contusion. J Trauma. 1988;28:298-304. 3351988 10.1097/00005373-198803000-00004

[10] Association for the Advancement of Automotive Medicine; Des Plaines I. The abbreviated injury scale 1990 update 1998. Accessed 12 September 2025. https://www.aaam.org/abbreviated-injury-scale-ais/

[11] Abdolrahimi SA , Sanati HR, Ansari-Ramandi MM, Heris SO, Maadani M. Acute myocardial infarction following blunt chest trauma and coronary artery dissection. J Clin Diagn Res. 2016;10:OD14-5. 10.7860/JCDR/2016/19043.7994 PMC4963698 27504338

[12] Ultee KHJ , Soden PA, Chien V, et al National trends in utilization and outcome of thoracic endovascular aortic repair for traumatic thoracic aortic injuries. J Vasc Surg. 2016;63:1232-1239.e1. 26776898 10.1016/j.jvs.2015.11.034 PMC4844787

[13] Shackford SR , Dunne CE, Karmy-Jones R, et al The evolution of care improves outcome in blunt thoracic aortic injury: a Western trauma association multicenter study. J Trauma Acute Care Surg. 2017;83:1006-1013. 28538630 10.1097/TA.0000000000001555

[14] Grigorian A , Spencer D, Donayre C, et al National trends of thoracic endovascular aortic repair versus open repair in blunt thoracic aortic injury. Ann Vasc Surg. 2018;52:72-78. 29886219 10.1016/j.avsg.2018.03.045

[15] Romagnoli AN , Dubose J. Is endovascular repair the first choice for all blunt aortic injury? A real-world assessment. J Cardiovasc Surg (Torino). 2019;60:289-297. 10.23736/S0021-9509.19.10909-3 30855117

[16] Procházka V , Roman J, Jalůvka F, et al Endovascular repair of thoracic aorta injury: 17 years of single-center experience. Med Sci Monit. 2021;27:e934479. 34759260 10.12659/MSM.934479 PMC8594114

[17] Schibilsky D , Driessen A, White WJ, et al Traumatic tracheobronchial injuries: incidence and outcome of 136.389 patients derived from the DGU traumaregister. Sci Rep. 2020;10:20555. 33239731 10.1038/s41598-020-77613-x PMC7688962

[18] Kiser AC , O'Brien SM, Detterbeck FC. Blunt tracheobronchial injuries: treatment and outcomes. Ann Thorac Surg. 2001;71:2059-2065. 11426809 10.1016/s0003-4975(00)02453-x

[19] Grewal HS , Dangayach NS, Ahmad U, Ghosh S, Gildea T, Mehta AC. Treatment of tracheobronchial injuries: a contemporary review. Chest. 2019;155:595-604. 30059680 10.1016/j.chest.2018.07.018 PMC6435900

[20] Bankhead-Kendall B , Radpour S, Luftman K, et al Rib fractures and mortality: breaking the causal relationship. Am Surg. 2019;85:1224-1227. 31775963

[21] Yeh DD , Hwabejire JO, DeMoya MA, Alam HB, King DR, Velmahos GC. Sternal fracture—an analysis of the national trauma data bank. J Surg Res. 2014;186:39-43. 24135374 10.1016/j.jss.2013.08.025

