# Supplementary material for: Prognostic Impact of Cardiovascular Injuries for Patients with Respiratory Isolated Chest Trauma
Source: Interdiscip Cardiovasc Thorac Surg. 2025 Nov 6;40(11):ivaf266. doi: 10.1093/icvts/ivaf266 (PMC12622769; doi:10.1093/icvts/ivaf266)
Supplement: ivaf266_Supplementary_Data [file ivaf266_supplementary_data.zip › Supplemental_Figure_S1.docx]

**Supplementary Figure S1A: Trend of in-hospital mortality among the 3 periods in the respiratory injury group**


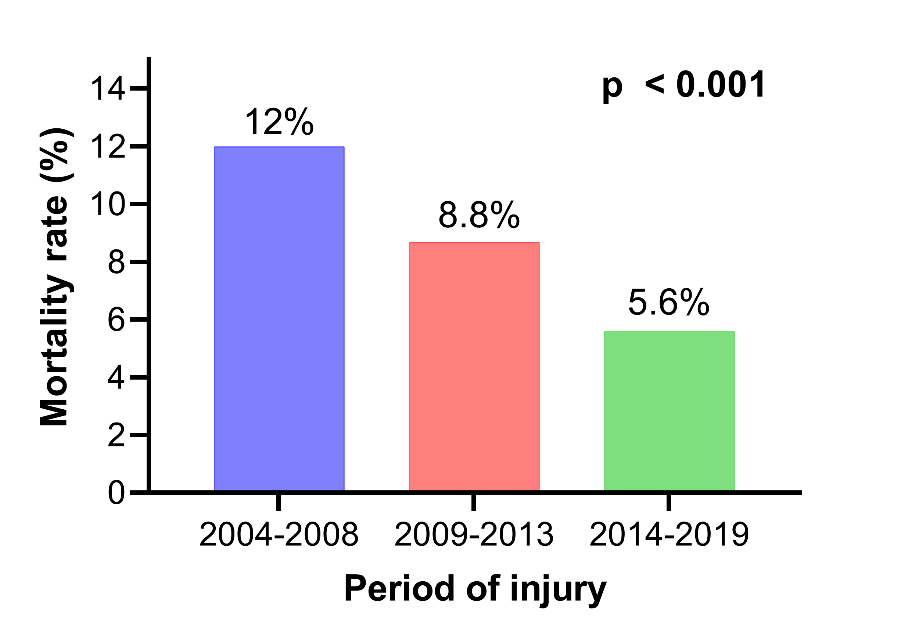


A significant decreasing trend is observed over time for in-hospital mortality in the respiratory injury group: 12% in 2004–2008, 8.8% in 2009–2013, and 5.6% in 2014–2019 (P < 0.001).

**Supplementary Figure S1B. Trend of in-hospital mortality among the 3 periods in the cardiovascular injury group**


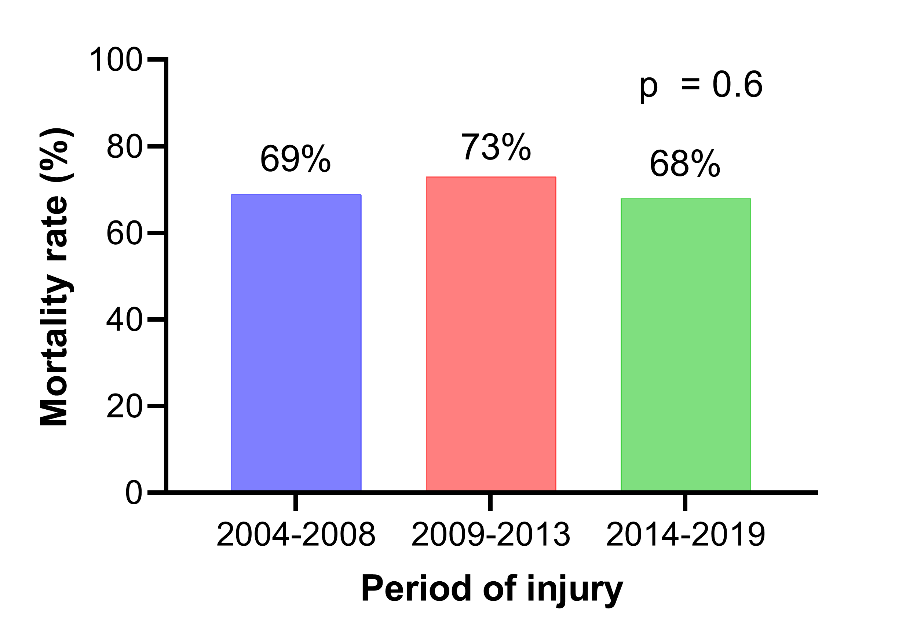


No significant trend is observed for in-hospital mortality in the cardiovascular injury group: 69% in 2004–2008, 73% in 2009–2013, and 68% in 2014–2019 (P = 0.6).
