# Supplementary material for: Prognostic Impact of Cardiovascular Injuries for Patients with Respiratory Isolated Chest Trauma
Source: Interdiscip Cardiovasc Thorac Surg. 2025 Nov 6;40(11):ivaf266. doi: 10.1093/icvts/ivaf266 (PMC12622769; doi:10.1093/icvts/ivaf266)
Supplement: ivaf266_Supplementary_Data [file ivaf266_supplementary_data.zip › Supplemntal_Table_S1_to_S2.docx]

| **Supplementary Table S1:** Specific variables in the Japan Trauma Data Bank | |
| --- | --- |
| **Variables** | **Description** |
| **Initial patient information** | |
| System ID | Unique number assigned to each case |
| Age |  |
| Sex |  |
| Onset | Including year, month, date, hour, and minute |
| Cause of trauma | Recorded as unexpected, suicide, assault, others, and unknown |
| Type of trauma | Recorded as penetrate, blunt, burn, others, and unknown |
| Transfer process | Means of transfer to the hospital including ambulance, helicopter, walk-in, private vehicle, others, and unknown |
| Finding time | Time of finding the scene, including year, month, date, hour, and minute |
| Arrival at scene | Time of arrival at scene, including year, month, date, hour, and minute |
| Contact at scene | Time of contact with patients, including year, month, date, hour, and minute |
| Leaving scene | Time of leaving scene, including year, month, date, hour, and minute |
| Arrival at hospital | Time of arrival at hospital, including year, month, date, hour, and minute |
| **Prehospital care** | Prehospital care such as oxygen, cervical collar, back board, cardiac massage, intubation, and intravenous line |
| **Findings at ambulance arrival** | |
| Vital signs | Including systolic pressure, diastolic pressure, heart rate, and respiratory rate |
| Japan Coma Scale |  |
| **Findings at hospital arrival** | |
| Vital signs | Including systolic pressure, diastolic pressure, heart rate, and respiratory rate |
| Temperature |  |
| Glasgow Coma Scale | Descripting its component, including E, V, and M, respectively |
| Japan Coma Scale |  |
| Alcohol consumption |  |
| **Examination information** | |
| Starting physician approach | Time of examination by physician, including year, month, date, hour, and minute |
| Existence of abnormal findings in ultrasound scan |  |
| CT examination | Recorded for each anatomical site with time including year, month, date, hour, and minute |
| Emergent angiography | Recorded for each anatomical site |
| **Treatment information** |  |
| Blood transfusion within 24 hours | Including year, month, date, hour, and minute |
| Initial operation | Including year, month, date, hour, and minute. Details are written in the supplemental source. |
| Re-operation within 48 hours after initial operation |  |
| **Information after admission** |  |
| Date of admission | Including year, month, date, hour, and minute |
| Department | Department of admission after initial management |
| Date of Emergency discharge | Including year, month, date, hour, and minute |
| Place after discharge | Recorded as home, another hospital, dead, others, and unknown |
| Date of death | Including year, month, date, hour, and minute |
| **Institute ID** |  |
| **Prefecture ID** |  |
| **Diagnosis and injury severity** |  |
| Injury Severity Score |  |
| cGCS | Weighting of the Glasgow Coma Scale in the calculation of the RTS |
| cBP | Weighting of the blood pressure in the calculation of the RTS |
| cRR | Weighting of the respiratory rate in the calculation of the RTS |
| RTS |  |
| cAge | Weight of age in the calculation of the predicted survival rate using the TRISS method |
| TRISS Ps | Predicted survival calculated by the TRISS method using coefficients derived from the Major Trauma Outcome Study |
| MAX AIS scores in body regions | Maximum injury severity in each of the 9 AIS body regions |
| Survival | Recorded as dead, survival, and unknown |
| **Mechanism of injury** | Details recorded individually |
| **Injured regions and AIS score** | Details recorded as supplemental source |
| **Emergency procedures** | Details recorded as supplemental source |
| **Information of initial operations** | Details recorded as supplemental source |
| **Medical history** | Details recorded as supplemental source |

AIS, Abbreviated Injury Scale; ID, identification; MAX, maximum; RTS, Revised Trauma Score; TRISS, Trauma and Injury Severity Score.

| **Supplemental Table S2:** Interventions after admission in the respiratory injury and cardiovascular injury groups | | | | | |
| --- | --- | --- | --- | --- | --- |
| Interventions | Respiratory | | Cardiovascular injury group | |  |
|  | injury group | |  |  |  |
|  | *n* = 8,048 | | *n* = 817 | | *P*-value |
| **Blood transfusion within 24 hours** | |  |  |  | <0.001 |
| Yes | 461 | (6) | 280 | (34) |  |
| No | 7,587 | (94) | 537 | (66) |  |
| **Emergency procedures** |  |  |  |  |  |
| Cardiac massage | 470 | (6) | 673 | (82) |  |
| Open chest | 160 | (2) | 408 | (50) |  |
| Tracheal intubation | 699 | (9) | 539 | (66) |  |
| Central venous catheterization | 196 | (2) | 133 | (16) |  |
| Vasopressor use | 275 | (3) | 278 | (34) |  |
| Chest drainage | 2586 | (32) | 228 | (28) |  |
| Aortic cross-clamping | 89 | (1) | 189 | (23) |  |
| Transcatheter arterial embolization | 62 | (1) | 14 | (2) |  |
| Resuscitative endovascular balloon occlusion of the aorta | 5 | (0.1) | 19 | (2) |  |
| Pericardiocentesis | 3 | (0.1) | 45 | (6) |  |
| Pericardial window | 10 | (0.1) | 99 | (12) |  |
| Others | 166 | (2) | 2 | (0.2) |  |
| **Urgent surgery** |  |  |  |  | <0.001 |
| Yes | 701 | (9) | 481 | (59) |  |
| Damage control surgery | 2 | (0.3) | 0 | (0) |  |
| Tracheobronchial repair | 23 | (3) | 0 | (0) |  |
| Pulmonary repair | 119 | (17) | 32 | (7) |  |
| Fracture repair or fixation | 88 | (13) | 2 | (0.4) |  |
| Diaphragm repair | 39 | (6) | 3 | (0.6) |  |
| Hemostasis | 255 | (36) | 228 | (47) |  |
| Vessel repair | 8 | (1) | 38 | (8) |  |
| Vascular graft replacement | 1 | (0.1) | 15 | (3) |  |
| Pericardial repair | 0 | (0) | 31 | (6) |  |
| Myocardial repair | 0 | (0) | 120 | (25) |  |
| Others | 138 | (20) | 148 | (31) |  |
| Unknown | 257 | (37) | 60 | (13) |  |
| No | 7,283 | (90) | 336 | (41) |  |
| Unknown | 64 | (1) | 0 | (0) |  |
